# Effect of a novel house (Star home) and toilet design on domestic fly densities in rural Tanzania

**DOI:** 10.1186/s13071-025-06722-1

**Published:** 2025-03-14

**Authors:** Arnold S. Mmbando, Amos J. Ngonzi, Salum Mshamu, John Bradley, Thomas Chevalier Bøjstrup, Halfan S. Ngowo, Jakob Knudsen, Lorenz von Seidlein, Fredros O. Okumu, Steve W. Lindsay

**Affiliations:** 1 Environmental Health and Ecological Sciences, Ifakara Health Institute, Ifakara, Tanzania; 2 Department of Biosciences, Durham University, Durham, UK; 3 CSK Research Solutions, Mtwara, Tanzania; 4 Nuffield Department of Clinical Medicine, University of Oxford, Oxford, UK; 5 London School of Hygiene and Tropical Medicine, London, UK; 6 The Royal Danish Academy–Architecture, Design, Conservation, Copenhagen, Denmark; 7 Mahidol-Oxford Tropical Medicine Research Unit (MORU), Bangkok, Thailand; 8 School of Public Health, Faculty of Health Sciences, University of the Witwatersrand, Parktown, Republic of South Africa; 9 Institute of Biodiversity, Animal Health and Comparative Medicine, University of Glasgow, Glasgow, UK

**Keywords:** Diarrhoea, Domestic flies, House screening, Randomised controlled trial, Tanzania

## Abstract

**Background:**

Diarrhoeal disease is the third leading cause of death in children under 5 years old with domestic flies acting as important mechanical vectors of diarrhoeal pathogens. To assess the effectiveness of a novel house design, “Star home”, and improved toilets in reducing the abundance of domestic flies, potential carriers of diarrhoeal pathogens, a randomized controlled trial was carried out in rural Tanzania.

**Methods:**

Domestic fly populations were monitored in 28 randomly selected Star homes and 28 traditional thatched roofs and mud-walled houses over 2 years from January 2022 to December 2023. Flies were sampled in kitchens and toilets using baited-fly traps from 07.00 h to 17.30 h every 7 weeks. To assess the production of flies from toilets, traps were placed over drop holes to collect emerging flies. Duration of external door openings to the kitchens was recorded with data loggers.

**Findings:**

Of the 1527 flies collected, 76% were *Chrysomya putoria*, 16% *Musca domestica* and 8% *Sarcophaga* spp. In kitchen collections, there were 46% fewer *C. putoria* flies [adjusted mean rate ratio (RR) = 0.54] and 69% fewer *Sarcophaga* spp. (RR = 0.31) in Star homes compared to traditional houses. There was no difference in the abundance of *M. domestica* in the two study groups. In toilets, there was 49% fewer *C. putoria* (RR = 0.51), but no difference was observed for other domestic fly species. No flies emerged from Star home toilets compared with a mean of 4.2 flies/trap/day in traditional toilets. During the day, the external doors od Star homes were open for an average of 13.0 min/h less than in traditional houses.

**Conclusions:**

Star homes reduced the abundance of domestic flies, apart from houseflies, in the kitchen and there were fewer *C. putoria*, a putative vector of diarrhoeal diseases, in Star home toilets compared to traditional houses. Changing the design of buildings can contribute to a decline in domestic flies and may lead to a reduction in diarrhoeal diseases.

**Graphical Abstract:**

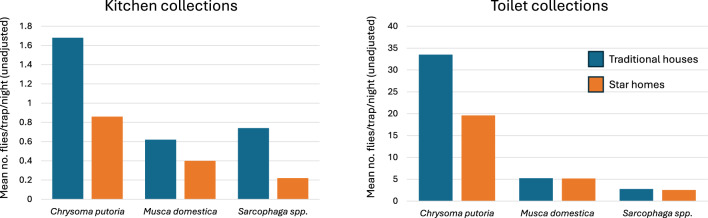

**Supplementary Information:**

The online version contains supplementary material available at 10.1186/s13071-025-06722-1.

## Background

Globally, diarrhoeal disease is the third leading cause of death in children under 5 years old, causing an estimated 443,832 deaths each year [1]. The major interventions used to prevent diarrhoea include safe water supplies, improved sanitation, hand washing with soap, exclusive breast feeding for the first 6 months of life, good personal and food hygiene, health education about how infections spread and rotavirus vaccination, but not the control of domestic flies.

Domestic flies such as *Musca domestica* (houseflies) and *Chrysomya putoria* (African latrine flies) are mechanical vectors of diarrhoeal pathogens [2–4]. Flies acquire pathogens through direct contact or ingestion of contaminated substances and subsequently transfer them to human food, utensils, or surfaces, thus facilitating the transmission of enteric pathogens [2]. Houseflies are particularly attracted to decaying food, faeces and garbage [5], which contribute to their role as mechanical vectors [6], transmitting pathogens like *Salmonella*, *Shigella* and toxic *Escherichia coli* [2]. Pit latrines are a major source of *C. putoria*, with one study in The Gambia demonstrating that peak production averages 500 flies/day [7]. A follow-up study in the same country showed that these flies also carried *Salmonella* spp., *Shigella* and toxic *E. coli* [3].

Previous studies showed that reducing adult fly abundance can reduce the transmission of diarrhoeal pathogens. In a prospective crossover intervention in Israeli army camps, the use of baited traps resulted in a 65% reduction in housefly counts, a 42% decrease in clinical diarrhoea visits and an 85% reduction in cases of shigellosis in the intervention bases compared to the controls [8]. A pilot study of indoor residual spraying with deltamethrin in The Gambia resulted in a 75% reduction in muscid flies and a 22% reduction in childhood diarrhoea cases during the wet seasons and a 26% reduction during the dry seasons compared to the control group [9].

Typically, fly control is based on environmental sanitation and hygiene reducing fly breeding sites, reducing sources of attraction, preventing contact between flies and diarrhoeal pathogens and protecting of food, eating utensils and people [10]. Surprisingly, little emphasis has been placed on the importance of house screening on reducing diarrhoeal diseases and fly contact.

An open-labelled randomized household trial was conducted to evaluate the impact of a novel-designed health house (Star homes) on reducing the incidence of childhood diarrhoea in the rural Mtwara district, Tanzania [11]. This article reports a secondary objective of the main trial: to reduce the abundance of domestic flies in Star homes compared to traditional houses. The present study was designed specifically to determine whether: (i) a Star home would reduce the number of domestic flies entering the kitchen compared with traditional houses and (ii) a novel-designed toilet, adjacent to the Star home, with a flap under the drop hole into the latrine, would reduce fly numbers in the toilet compared with traditional houses.

## Methods

### Study area

**Fig. 1:**
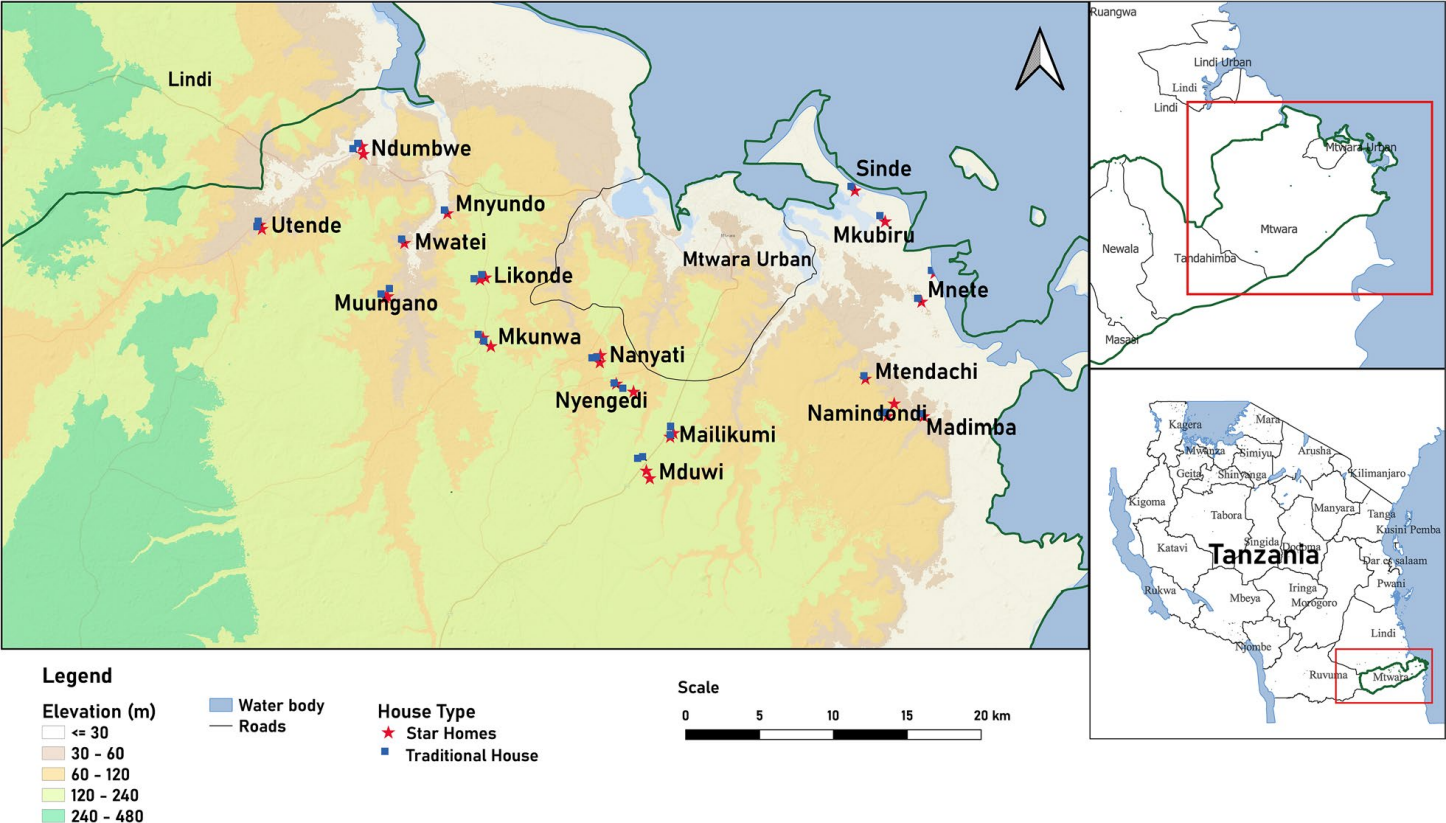
Location of study houses

The study was conducted in rural Mtwara district (Fig. 1), southeastern Tanzania, from January 2022 to December 2023. The study area consists of a coastal strip of sandy low-lying land and undulating hills inland, with elevation up to 400 m above sea level. The region is mainly covered by dense forests and shrublands. It typically experiences two distinct rainy seasons: a longer one from February to April and a shorter one from October to December. A range of dipteran families have been recorded in the area including Psychodidae, Culicidae, Calliphoridae, Syrphidae, Stratiomyidae and Sarcophagidae [12]. The prevalence of diarrhoea in children was 48% in 2009 and 37% in 2010 [13].

### Study design

A detailed description of the study design is provided by Mshamu et al. [11]. Briefly, this is a cohort, open-label household randomized control study. Surveillance of domestic flies with baited traps was carried out during the day in the kitchens and toilets of 28 Star homes and 28 traditional houses every 7 weeks from January 2022 to December 2023. Each Star home was paired with the nearest traditional house willing to participate in the study, and fly collections were made simultaneously in pairs of houses. The duration of door opening of the main external door leading into the kitchen was also recorded in study houses. The primary outcome measure was a comparison of the number of domestic flies collected in the kitchens and toilets using baited traps. The secondary outcome was a comparison of the duration of door opening in both study groups.

### Interventions

Star homes (Supplementary Fig. 1a) had four features that could potentially reduce the entry of domestic flies into the kitchen: (i) kitchen walls made of screened fly-proof netting (Supplementary Fig. 1b), (ii) self-closing and well-fitting external screened doors, (iii) a screened storage area for storing food and cooking utensils and (iv) a cement floor that allows efficient floor cleaning. However, in Star homes the wood-burning stove was fitted with a chimney to remove wood smoke and the kitchen was designed to be well ventilated by having fly-screened walls to reduce indoor air pollution. Since wood smoke is fly repellent [14], a smoke-free kitchen (Supplementary Fig. 2b) is unlikely to deter flies from the kitchen compared with traditional smoky kitchens (Supplementary Fig. 2c). There was also a behavioural element to the intervention with owners of Star homes asked to keep their self-closing doors closed. In traditional houses, kitchens are situated outdoors, approximately 2 m from the house. Kitchens are mostly open shelters, with a thatched roof and open sides.

Each Star home had a toilet (Supplementary Fig. 2a) situated 10 m from the house, with cement floors for easy cleaning, was well-ventilated and had a flap under the drop hole (Supplementary Fig. 2b), preventing entry and exit of flies. In contrast, traditional toilets are fenced structures without a roof (Supplementary Fig. 2c). The cover over the pit is commonly made from wattle and mud daub, which is difficult to clean and has no cover over the drop holes (Supplementary Fig. 2d), allowing flies access into and out of the toilet.

### Inclusion criteria for study households

Traditional houses were eligible for inclusion in the study if they had: (i) mud walls, a thatched roof and a dirt floor, (ii) a toilet, (iii) absence of grid electricity, (iv) no access to piped water, (v) sufficient land for Star home construction, (vi) residence of at least two children < 13 years old and (vii) willingness of occupants to participate in 3 years of disease surveillance.

### Randomization and masking

In the main study, 110 Star homes were constructed with 440 traditional houses enrolled in the study [11]. In the present study, a sub-sample of 28 Star homes and 28 traditional houses was randomly selected from the 110 traditional houses used for routine mosquito collections (Mmbando et al., in preparation). The number of houses sampled was reduced for logistical reasons. For each Star home, the nearest traditional house enrolled in the study was selected, with at least two Star homes and four traditional houses sampled in each village. The same 56 houses were visited every 7 weeks for 2 years. Not all study procedures were fully masked, but bias was reduced using fly traps independent of fieldworker skill. Different technicians, unaware of trap locations, analysed catches.

### Study procedures

#### Observational survey

Repeated observations were conducted in all 56 study houses every 7 weeks. Daytime inspections of kitchens and toilets took place immediately after trap setting between 08.00 h and 10.00 h. Fieldworkers, with one assigned to each pair of houses (one Star home and one traditional house), utilized a structured questionnaire on a Samsung Galaxy Tab A7 Lite tablet, employing KOBO-collect software. The questionnaires recorded information on kitchen use and toilet locations, designs and building materials.

To assess cooking practices, mothers were interviewed about where they cooked and their reasons for cooking indoors or outdoors. This was supported by observations made by the fieldworkers on the presence of ash in stoves and firewood. For households cooking outdoors, fly traps were placed inside the house near cooking utensils and leftover food. For toilets, data were collected on construction materials, toilet design and the presence or absence of holes in the latrine cover in addition to the main drop hole.

#### Domestic fly trapping

Odour-baited fly traps [15] were used for collecting domestic flies (Supplementary Fig. 3). Each trap consisted of a 3 L rectangular polypropylene box (Whitefurze, Coventry, UK, Supplementary Fig. 3a) with a white opaque lid containing 5 circular entrance holes, each 6–8 mm in diameter (Supplementary Fig. 4b). In the box, 50 g of saltwater fish was placed in a 9-cm-diameter, 6-cm-high plastic bowl (W.K. Thomas, Chessington, UK) covered with untreated cotton netting.

Each study house had two traps: one in the kitchen at the furthest corner from the main door (Supplementary Figs. 5a and c) and another in the toilet at the furthest corner from the door (Supplementary Fig. 5b and d). Since many traditional toilets had no roof, the traps were vulnerable to flooding in heavy rain. Therefore, traps were raised 20 cm above the ground on a metal frame, and a small roof was constructed over the trap (Supplementary Fig. 3c). Each week, 16 odour-baited fly traps were positioned in the kitchens and toilets of both house types. Traps were positioned in the corner of the kitchen and toilets farthest from the main entrance from 07.00 h to 17.30 h on the same day.

Emergence of flies from toilets was measured using funnel traps in Star home toilets (Supplementary Fig. 5a) and traditional toilets (Supplementary Fig. 5b). Traps for toilets were rectangular at the base, 50 cm long, 25 cm wide and 60 cm high, constructed from a metal frame, covered with untreated mosquito netting and placed directly over the drop hole from 10.00–12.00 h. Mechanical aspirators (Prokopack^®^, model 1419, John W. Hock Co., Gainesville, FL, USA) [16] were used to collect flies from the traps. Specimens were packed into labelled nets and transported to the field laboratory for sorting and data recording. Sampling was carried out weekly for 5 months (from 30 March 2023 to 11 August 2023).

#### Door opening and closing

Duration of door opening was recorded in eight houses per week/cluster, comprising four Star homes and four traditional houses. For each house type, one door logger (Onset UX90-001-HOBO/state/pulse) was installed on the inner side of the main door leading into the kitchen. The data loggers recorded the duration of the door opening every 30 s from 06.00 h to 18.00 h.

### Statistical analysis

Data were analysed using R language version 3.5.0 and followed an analysis plan written before study completion. The primary outcome measures were: (i) the number of domestic fly species collected in Star home kitchens compared to kitchens of traditional houses and (ii) the number of flies collected in Star home toilets compared with traditional toilets. All analyses were conducted on an intention-to-treat basis, i.e. regardless of whether families actively used the kitchen or not. In addition, we hypothesized that in both study groups kitchens in use would attract more flies than kitchens not used for cooking because of the presence of food and dirty cooking utensils. Therefore, we compared fly abundance between Star home households actively using their kitchens and those that were not.

Domestic fly counts were modelled using a Generalized Linear Mixed Model (GLMM) with the *glmmTMB* package [17], employing a negative binomial distribution to account for over-dispersion. The response variable was daily fly counts per house, and the main fixed variable was the interventions applied. Nested random terms were added to account for variations across days, villages, house pairs, rounds, and clusters. Each domestic fly species was analysed separately. Additionally, GLMMs were used to assess fly abundance by species and season between Star home families who use kitchens and those who do not and in traditional houses where cooking occurs outdoors or indoors (bedroom or living room). Fly counts per species were treated as the response variable and kitchen use (yes/no) as the fixed factor, with random factors including round, village and house ID pairs.

Data on duration of door opening were summarized into hours of the day, from 06.00 h to 18.00 h, when flies are likely to be most active. A linear mixed-effects model (*lmer*) with a normal distribution determined the adjusted daily mean differences in door opening durations across traditional and Star homes, including their 95% confidence intervals.

## Results

### Observational study

A description of the study kitchens and their use is shown in Supplementary Table 1. In Star homes, fewer people cooked indoors during the dry season than in the wet season. Star home kitchens were uniform; those in traditional houses were either indoors in rooms also used as living quarters or bedrooms. Half of respondents (50%, 173/348) reported that several factors hindered them from using the Star home kitchen, including the smaller diameter of the cooking fire 52% (90/173), smoke in the kitchen 30% (52/173) and a reluctance to change from cooking in the traditional manner 18% (31/173).

### Kitchen collections

During the study, 1527 domestic flies were collected from 680 trapping occasions in kitchens, 32% (494/1527) in Star homes and 68% (1033/1527) in traditional houses. Of these, 56.3% were *C. putoria* (859/1527), 22.4% *M. domestica* (342/1527) and 21.3% *Sarcophaga* spp. (326/1527). Overall, Star home kitchens had 46% fewer *C. putoria* (*p* = 0.002) and 69% fewer *Sarcophaga* spp. (*p* < 0.0001) than traditional houses, although there was no significant reduction in *M. domestica* between the study groups (*p* = 0.114, Table 1). There was no difference in fly numbers collected in the dry and wet seasons (Supplementary Table 2). In all comparisons, there was no difference in fly abundance between those who used Star home kitchens and those who did not (Supplementary Table 3) or between those in traditional houses who cooked indoors or outdoors (Supplementary Table 4).

**Table 1:** Domestic fly abundance in study house kitchens at different times of the year

Species	House type	No. trap collections	Total caught	Unadjusted mean (95% CI)	Adjusted mean (95% CI)	Adjusted RR (95% CI)	% reduction	*p*
Dry season (June–November)								
* Chrysomya putoria*	Traditional house	187	208	1.11 (0.68–1.54)	0.68 (0.29–1.60)	1		
	Star homes	187	96	0.53 (0.31–0.75)	0.31 (0.13–0.75)	0.46 (0.26–0.80)	54	0.005
* Musca domestica*	Traditional house	187	39	0.21 (0.10–0.32)	0.10 (0.02–0.45)	1		
	Star homes	187	21	0.11 (0.05–0.19)	0.05 (0.01–0.23)	0.50 (0.21–1.20)	50	0.122
* Sarcophaga species*	Traditional house	187	115	0.62 (0.32–0.92)	0.36 (0.16–0.81)	1		
	Star homes	187	41	0.23 (0.09–0.37)	0.12 (0.04–0.33)	0.34 (0.15–0.73)	66	0.006
Wet season (December–May)								
* Chrysomya putoria*	Traditional house	153	363	2.37 (1.47–3.27)	1.55 (0.61–3.95)	1		
	Star homes	153	192	1.25 (0.85–1.65)	1.02 (0.50–2.09)	0.65 (0.37–1.16)	25	0.05
* Musca domestica*	Traditional house	153	174	1.11 (0.40–1.82)	0.42 (0.13–1.35)	1		
	Star homes	153	111	0.73 (0.37–1.09)	0.32 (0.10–1.01)	0.85 (0.42–1.71)	15	0.650
* Sarcophaga species*	Traditional house	153	138	0.90 (0.53–1.27)	0.23 (0.04–1.81)	1		
	Star homes	153	32	0.21 (0.12–0.30)	0.06 (0.01–0.33)	0.26 (0.14–0.50)	74	< 0.0001
Dry and wet seasons (January 2022–December 2023)								
* Chrysomya putoria*	Traditional house	340	571	1.68 (1.21–2.15)	0.95 (0.49–1.86)	1		
	Star homes	340	288	0.86 (0.64–1.08)	0.51 (0.26–1.01)	0.54 (0.36–0.80)	46	0.002
* Musca domestica*	Traditional house	340	209	0.62 (0.29–0.95)	0.22 (0.08–0.62)	1		
	Star homes	340	133	0.40 (0.23–0.57)	0.14 (0.04–0.41)	0.64 (0.37–0.11)	36	0.114
* Sarcophaga species*	Traditional house	340	253	0.74 (0.51–0.97)	0.33 (0.15–0.71)	1		
	Star homes	340	73	0.22 (0.13–0.31)	0.10 (0.04–0.23)	0.31 (0.18–0.53)	69	< 0.0001

*RR* = risk ratio, *CI* = 95% confidence intervals, *p* = probability

### Toilet collections

Among the 28 traditional houses included in the study, 82% (23/28) had toilets, resulting in the exclusion of the remaining houses where inhabitants practiced open defaecation from the analysis. The toilets in traditional houses were primarily constructed with walled thatch and grass mounted on wooden sticks with earth floors. Approximately 80% of these toilets lacked roofs, and over 70% had doorways covered by curtains (Supplementary Table 1).

A total of 23,211 domestic flies were collected over 672 trapping occasions, where 40% (9164/23211) were from Star home toilets and 60% (14,047/23211) from traditional toilets. Of these, 77% were *C. putoria* (17,920/23,211), 15% *M. domestica* (3507/23,211) and 8% *Sarcophaga* spp. (1784/23,211). Overall, Star home toilets had 51% fewer *C. putoria* species than traditional houses (*p* < 0.001; Table 2), although there was no difference between the numbers of *Sarcophaga* spp. (*p* = 0.495) or *M. domestica* (*p* = 0.198) collected between the study groups. There were 54% more *C. putoria* in toilets of both house types during the wet season than the dry season (RR = 1.54, 95% CI 0.75–1.73, *p* = 0.041, Supplementary Table 2).

**Table 2:** Domestic fly abundance in study house toilets at different times of the year

Domestic fly species	House type	No. trap collections	Total caught	Unadjusted mean (95% CI)	Adjusted mean (95% CI)	Adjusted RR (95% CI)	% reduction	*p*
Dry season (June–November)								
* Chrysomya putoria*	Traditional house	182	4610	24.8 (19.89–29.71)	20.21 (12.11–33.74)	1		
	Star homes	182	2390	13.1 (9.64–16.56)	9.57 (5.69–16.08)	0.47 (0.35–0.63)	53	< 0.001
* Musca domestica*	Traditional house	182	236	1.27 (0.86–1.68)	1.06 (0.61–1.85)	1		
	Star homes	182	224	1.23 (0.76–1.70)	0.91 (0.52–1.61)	0.86 (0.55–1.34)	14	0.501
* Sarcophaga species*	Traditional house	182	349	1.88 (1.46–2.30)	1.87 (1.34–2.60)	1		
	Star homes	182	379	2.08 (1.74–2.42)	1.97 (1.41–2.74)	1.06 (0.62–1.15)	6	0.707
Wet season (December–May)								
* Chrysomya putoria*	Traditional house	154	6731	44.0 (34.21–53.79)	35.87 (15.30–84.11)	1		
	Star homes	154	4189	27.2 (21.25–33.15)	17.73 (7.57–41.48)	0.49 (0.36–0.69)	41	< 0.001
* Musca domestica*	Traditional house	154	1536	10.0 (6.02–13.08)	2.90 (1.00–8.42)	1		
	Star homes	154	1511	9.81 (6.29–13.33)	2.24 (0.77–6.48)	0.78 (0.53–1.12)	22	0.175
* Sarcophaga species*	Traditional house	154	585	3.82 (2.78–4.86)	2.78 (1.32–5.86)	1		
	Star homes	154	471	3.06 (2.33–3.79)	2.30 (1.01–4.85)	0.83 (0.59–1.15)	17	0.263
Dry and wet seasons (January 2022–December 2023)								
* Chrysomya putoria*	Traditional house	336	11,341	33.5 (28.23–38.77)	24.96 (15.25–40.86)	1		
	Star homes	336	6579	19.6 (16.21–22.99)	12.72 (7.74–20.90)	0.51 (0.41–0.63)	49	< 0.001
* Musca domestica*	Traditional house	336	1772	5.23 (3.75–6.71)	1.89 (0.89–4.00)	1		
	Star homes	336	1735	5.16 (3.47–6.85)	1.55 (0.73–3.29)	0.82 (0.61–1.11)	19	0.198
* Sarcophaga species*	Traditional house	336	934	2.76 (2.23–3.29)	2.22 (1.42–3.47)	1		
	Star homes	336	850	2.53 (2.14–2.92)	2.05 (1.31–3.21)	0.93 (0.74–1.16)	7	0.495

*RR* = risk ratio, *CI* = 95% confidence intervals, *p* = probability

### Exit trap collections from toilets

During this sub-study, we collected flies directly emerging from the drop holes on 104 trapping occasions: 52 from Star homes and 52 from traditional house toilets. No flies were caught in exit traps in Star home toilets compared to 219 flies from traditional toilets. Of the flies collected, 72% (158/219) were *C. putoria*, 24% (52/219) *M. domestica* and 4% (9/219) *Sarcophaga* spp.

### Duration of main door openings

**Fig. 2:**
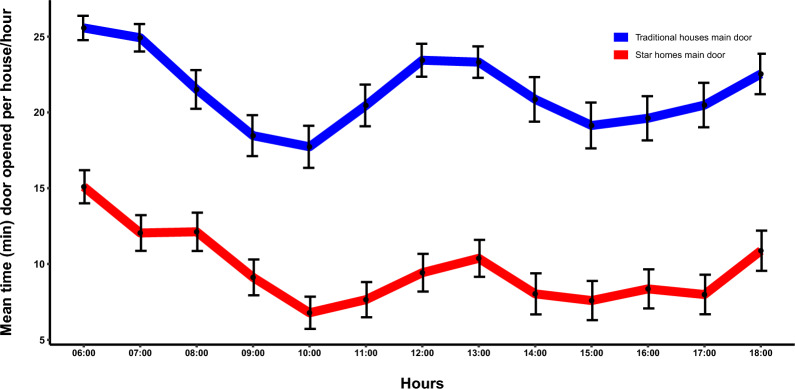
Kitchen door openings in Star homes (red line) and traditional houses (blue line). Error bars are (95% CIs)

The pattern of door opening during daylight hours was similar in both study groups (Fig. 2). Duration of door opening was greatest in the early morning at 06.00 h declining steadily until 10.00 h. There was a second peak at midday, from 12.00 to 13.00 h, followed by a further decline and then a gradual rise from 15.00 to 18.00 h. The external kitchen doors of Star homes (mean opening time = 7.4 min, 95% CI 4.5–10.2) were open for shorter periods than the kitchen doors of traditional houses (mean opening time = 20.7 min, 95% CI: 18–23.4). Star home kitchen doors remained open for 67% less time (equivalent to 14 min/h) than in traditional houses, with an adjusted mean difference of – 13.3 min per hour (95% CI: − 16.5, − 10.2; *p* < 0.0001; Table 3).

**Table 3:** Duration of the main door openings (min/h) with 95% confidence intervals (CI)

House typology	No. trap collections	Unadjusted mean (min) (95% CI)	Adjusted mean difference (min) (95% CI)	*p*
Dry seasons (June–November)				
Traditional house	84	20.0 (17.0, 23.0)	1	Ref
Star homes	84	6.2 (3.1, 9.4)	−13.7 (−17.2, −10.2)	< 0.00001
Wet seasons (December–May)				
Traditional house	76	21.3 (17.4, 25.2)	1	Ref
Star homes	76	10.6 (6.7, 14.4)	−10.7 (−15.4, −6.1)	< 0.0001
Dry and wet seasons (February 2022–December 2023)				
Traditional house	160	20.7 (18.0, 23.4)	1	Ref
Star homes	160	7.4 (4.5, 10.2)	−13.3 (−16.5, −10.2)	< 0.0001

Mean values were adjusted for house type, rounds and month of collections. *p* = probability

## Discussion

Preventing the interaction between humans and domestic flies, which serve as mechanical vectors for diarrhoeal pathogens, is important for reducing the incidence of childhood diarrhoea [9]. This study demonstrated the effectiveness of Star homes in reducing domestic fly populations in both the kitchen and toilets compared to traditional houses. During the 2-year study, there were 46% fewer *C. putoria* and 69% fewer *Sarcophaga* species in the kitchens of Star homes compared to traditional houses. This is likely to have positive health impacts since *C. putoria* is a putative vector of diarrhoeal diseases [3] and *Sarcophaga* species can transmit bacteria (*Bacillus anthracis* and *Pseudomonas auregunosa* [18]) and fungi (*Aspergillus* species [19]), and their larvae cause myiasis. Although there were 36% fewer *M. domestica* in Star home kitchens than in traditional kitchens, this did not reach statistical significance, perhaps because of the low numbers caught. Alternatively, the reduced protective efficacy of Star home kitchens in reducing *M. domestica* may be due to the flies' strong preference for proximity to humans, which increases their chances of entering houses [4]. There was no difference in domestic fly abundance in the kitchens of either house type between dry and wet seasons, indicating that Star homes' protection against indoor fly entry was unaffected by seasonal changes. Fly numbers did not differ when people cooked in indoor or outdoor kitchens.

A number of design features of the Star home kitchens likely contributed to the reduction in domestic flies; however, our design did not make determining which interventions were most important possible. The shade netting on the walls acted as physical barriers, preventing domestic flies from entering the kitchens, as observed in previous studies [20]. The self-closing solid doors in the intervention house kitchens also acted as physical barriers against the flies and ensured the doors stayed closed for longer than in traditional houses. Keeping the doors closed for longer periods probably further increased the protective efficacy of Star homes against house entry by domestic flies. Lastly, the smooth cemented floors in the intervention houses facilitated easier cleaning in contrast to the frequently encountered porous and earthen floors in traditional houses. This ensured efficient removal of all food materials dropped during cooking, thereby reducing the presence of decaying food odours, which attracts flies to the kitchen. In contrast, in the traditional house kitchens with earth floors, food spilled on the floor is absorbed into the ground and smells persist. In the Star homes, respondents were also regularly reminded to keep their doors closed.

Overall, around 49% of families in both Star homes and traditional houses cooked outdoors during the dry season, which decreased to 25% in the rainy season. A systematic review of the literature showed that complete and immediate acceptance of a new mode of cooking is unusual [21]. Often people use multiple options, referred to as “stacking”, where users stack alternative fuels or cooking methods as a back-up to the new intervention. In the present study, we identified several ways to improve the characteristics of the stove in Star home kitchens [22] including increasing the size of the stove to enable cooking large meals (e.g. during festivals) and for several hours (e.g. when cooking *Ugali*). Most householders referred to this problem and were thus falling back on the traditional three-stone stoves used outside the house. Based on these lessons, we propose a stove design with a larger diameter, preferably with options to modify the size of the dish holder depending on the type of cooking as well as a larger chamber for fuel intake and burning.

During the day, the main doors to Star homes were open for 14 min/h less than the main doors of traditional houses. The general patterns seen reflect how people use the house. High activity was observed at sunrise (06.00 h), coinciding with people waking up, gradually diminishing until 10.00 h, when both men and women are engaged in farm work, children are heading to school, and household cleaning is underway. A subsequent increase in activity occurs, reaching a peak at 13.00 h, corresponding with householders preparing and consuming their lunch. Following a subsequent decline in door opening, there was a gradual increase after 15.00 h, as individuals returned home from the farms and children return from school. Households were involved in domestic activities such as fetching water, washing dishes and preparing their evening meal.

Star home toilets had roughly half the number of *C. putoria* compared with traditional toilets. This is an important finding since this species represented 77% of the total flies trapped in the toilets and is a putative vector of diarrhoea pathogens [3] since it breeds in faeces contained within the toilet. This reduction can be attributed to the flap under the drop hole, which prevents flies from entering or exiting the faecal waste chamber despite higher fly numbers caught in wet seasons. This conclusion is supported by findings from trap collections directly over the drop hole, which caught no flies in Star home toilets compared with 219 flies exiting from traditional toilets that lacked a protective cover over the drop hole. It follows, therefore, that *C. putoria* flies collected in Star home toilets originated from the surrounding environment, particularly in villages, where open defaecation is common. The numbers of *C. putoria* collected in toilets rose during the wet season, presumably since wet toilets were more conducive to an increase in adult fly production from traditional toilets as well as increasing adult fly survival.

The study had two main limitations. First, since Star homes had fewer exit points for flies than traditional houses, it is probable that traps in the Star homes captured a higher proportion of house-entering flies compared to those in traditional houses. Second, a similar source of bias was expected when sampling flies within the Star home toilets. Both limitations would underestimate the true efficacy of our interventions in capturing flies.

The cost of a Star home and ways to finance their building will be the subject of a future publication by the research team.

## Conclusions

Star homes effectively reduced the abundance of *C. putoria*, a putative vector of diarrhoeal pathogens, and *Sarcophaga* spp., a cause of myiasis, in their kitchens compared to traditional houses. The toilets in Star homes reduced the most common fly, *C. putoria*, but had no effect on other domestic fly species compared to traditional toilets. These results show how well-screened houses and toilets which prevent flies entering the faecal collecting pit can reduce the abundance of important vectors of diarrhoeal diseases. Even greater reductions would probably occur if the quality of housing and toilets were improved on a larger scale. Our findings are of relevance to those designing and constructing new homes in sub-Saharan Africa.

## Supplementary Information

Additional file 1.

## Data Availability

Data sharing (deidentified) is available to researchers whose proposed purpose of use is approved by the Mahidol University Oxford Tropical Medicine Research Unit data access Articles 90 www.thelancet.com/infection Vol 23 January 2023 committee. Related documents such as the study protocol and informed consent form will be made available on request. To request the dataset, please send a signed data request form to datasharing@tropmedres.ac. The data request form can be found online at https://www.tropmedres. ac/units/moru-bangkok/bioethics-engagement/data-sharing.

## Abbreviations

CI: Confidence intervals
*C. putoria*: *Chrysomya putoria*
GLMM: Generalized linear mixed effect model
Lmer: Linear-mixed effect model
*M. domestica*: *Musca domestica*
n/a: Not applicable
p: Statistical probability
TMB: Template model builder
spp.: Species
