# Supplementary material for: Effect of a novel house (Star home) and toilet design on domestic fly densities in rural Tanzania
Source: Parasit Vectors. 2025 Mar 14;18:106. doi: 10.1186/s13071-025-06722-1 (PMC11908010; doi:10.1186/s13071-025-06722-1)
Supplement: Supplementary file 1 — Additional file 1. [file 13071_2025_6722_MOESM1_ESM.docx]

**Supplementary material**

**Supplementary Table 1** Observational study of kitchens and toilets in the Star homes and traditional houses

| **Characteristic** | **Star homes (% n/N)** | **Traditional houses (% n/N)** |
| --- | --- | --- |
| **Indoor kitchen** | | |
| Building structure | Shade cloth mounted on steel frames with concrete floor (100%, 28/28) | Mud and stick walls roofed by the thatched roof (100% 15/15) |
| Mean dimensions (m) | 3 m wide x 3.6 m long x 2.4 m high | 2 m wide x 3.5 m long x 2 m high |
| Position/location of the kitchen | Separate kitchen area (100%, 28/28) | Living room (60%, 9/15)  Bedrooms (40%, 6/15) |
| Occasions used in dry season | 35%, 65/188 | 67%, 126/188 |
| Occasion used in the wet season | 69%, 110/160 | 81%, 129/160 |
| **Outdoor kitchen** | | |
| Structure | n/a | Completely open kitchen structure (62%, 8/13), thatched wall mounted on wooden poles (31%, 4/13), metal sheet walls nailed on wooden poles (8%, 1/13) & unroofed kitchen structures (62%, 8/13). |
| Mean dimensions (m) | - | 1.4 m wide x 2 m long x 1.5 m high |
| Occasions used in the dry season | 65%, 123/188 | 33%, 62/188 |
| Occasion used in the wet season | 31%, 50/160 | 19%, 31/160 |
|  |  |  |
| Building structure | Concrete cladding walls on a steel frame with concrete floor frames with concrete floor (100%, 28/28) | Grass or thatched walls tighten on wooden sticks with earth floors (100%, 23/23) |
| Mean dimensions (m) | - | 1.2m width * 2m length * 1.3m height |
| Door cover | Metallic door (100%, 28/28) | Curtains (62%, 16/23), completely open doorway (17%, 4/23), sticks and grass (13%, 3/23) |
| Roof | Corrugated metal sheets (100%, 28/28) | Thatched or grass roofed (17%, 4/23), unroofed (83%,19/23) |

**Supplementary Table 2** Domestic fly abundance caught in the kitchens and toilets in the dry and wet the seasons. Where, RR=Risk ratio, CI=95% Confidence intervals, p= probability.

| **Species** | **Season** | **No. trapping occasions** | **Total caught** | **Unadjusted Mean**  **(95% CI)** | **Adjusted RR**  **(95% CI)** | **p** |
| --- | --- | --- | --- | --- | --- | --- |
| **Kitchen** | | | | | | |
| *Chrysomya putoria* | Dry | 376 | 304 | 0.81 (0.54 – 1.05) | 1 |  |
|  | Wet | 320 | 320 | 2.04 (1.29 – 2.79) | 1.87 (0.87 – 4.02) | 0.106 |
| *Sarcophaga* spp | Dry | 376 | 156 | 0.42 (0.22 – 0.62) | 1 |  |
|  | Wet | 320 | 187 | 0.58 (0.39 – 0.77) | 0.72 (0.32 – 1.66) | 0.444 |
| *Musca domestica* | Dry | 376 | 60 | 0.16 (0.10 – 0.22) | 1 |  |
|  | Wet | 320 | 299 | 0.98 (0.54 – 1.32) | 2.13 (0.68 – 6.69) | 0.197 |
| **Toilets** | | | | | | |
| *Chrysomya putoria* | Dry | 376 | 7004 | 18.70 (15.68 – 21.72) | 1 |  |
|  | Wet | 320 | 11434 | 35.70 (13.30 – 37.59) | 1.54 (1.02 – 2.32) | 0.041 |
| *Sarcophaga* spp | Dry | 376 | 733 | 1.95 (1.68 – 2.22) | 1 |  |
|  | Wet | 320 | 1140 | 3.56 (2.92 – 4.20) | 1.14 (0.75 – 1.73) | 0.568 |
| *Musca domestica* | Dry | 376 | 460 | 1.23 (0.92 – 1.59) | 1 |  |
|  | Wet | 320 | 3067 | 9.58 (7.33 – 11.83) | 1.48 (0.76 – 2.86) | 0.248 |

**Supplementary Table 3** Domestic fly abundance in the Star homes families who utilise the kitchen versus non-users. Where, RR=Risk ratio, CI= 95% Confidence intervals and p= probability.

| **Species** | **Kitchen use status** | **Total number of collections** | **Total caught** | **Unadjusted Mean (95% CI)** | **Adjusted RR**  **(95% CI)** | **p** |
| --- | --- | --- | --- | --- | --- | --- |
| **Dry seasons (June – November)** | | | | | | |
| *Chrysomya putoria* | Non-user | 123 | 72 | 0.6 (0.3 – 0.9) | 1 |  |
|  | User | 65 | 24 | 0.4 (0.1 – 0.6) | 0.76 (0.29 – 1.99) | 0.58 |
| *Sarcophaga* species | Non-user | 123 | 33 | 0.3 (0.1 – 0.4) | 1 |  |
|  | User | 65 | 8 | 0.1 (0.0 – 0.3) | 0.28 (0.04 – 2.21) | 0.23 |
| *Musca domestica* | Non-user | 123 | 9 | 0.1 (0.0 – 0.2) | 1 |  |
|  | User | 65 | 12 | 0.2 (0.0 – 0.4) | 2.45 (0.58 – 10.27) | 0.22 |
| **Rainy seasons (December – May)** | | | | | | |
| *Chrysomya putoria* | Non-user | 50 | 76 | 1.5 (0.8 – 2.3) | 1 |  |
|  | User | 110 | 116 | 1.1 (0.5 – 1.6) | 0.61 (0.26 – 1.41) | 0.25 |
| *Sarcophaga* species | Non-user | 50 | 12 | 0.2 (0.0 – 0.4) | 1 |  |
|  | User | 110 | 22 | 0.2 (0.1 – 0.3) | 1.08 (0.34 – 3.44) | 0.89 |
| *Musca domestica* | Non-user | 50 | 40 | 0.8 (0.2 – 1.3) | 1 |  |
|  | User | 110 | 73 | 0.7 (0.3 – 1.1) | 0.45 (0.13 – 1.53) | 0.20 |
| **Dry & Wet seasons (January 2022 – December 2023)** | | | | | | |
| *Chrysomya putoria* | Non-user | 173 | 148 | 0.9 (0.6 – 1.2) | 1 |  |
|  | User | 175 | 140 | 0.9 (0.6 – 1.2) | 0.77 (0.41 – 1.46) | 0.42 |
| *Sarcophaga* species | Non-user | 173 | 45 | 0.3 (0.2 – 0.4) | 1 |  |
|  | User | 175 | 30 | 0.2 (0.1 – 0.3) | 0.61 (0.23 – 1.60) | 0.32 |
| *Musca domestica* | Non-user | 173 | 49 | 0.3 (0.1 – 0.5) | 1 |  |
|  | User | 175 | 85 | 0.5 (0.2 – 0.8) | 1.06 (0.38 – 2.91) | 0.91 |

**Supplementary table 4** Domestic fly abundance in traditional houses with indoor versus outdoor cooking. Where, RR=Risk ratio, CI= 95% Confidence intervals and p= probability.

| **Species** | **Kitchen use status** | **Total number of collections** | **Total caught** | **Unadjusted Mean (95% CI)** | **Adjusted RR**  **(95% CI)** | **p** |
| --- | --- | --- | --- | --- | --- | --- |
| **Dry seasons (June – November)** | | | | | | |
| *Chrysomya putoria* | Outdoor | 62 | 65 | 1.1 (0.4 – 1.8) | 1 |  |
|  | Indoor | 126 | 143 | 1.1 (0.6 – 1.6) | 1.67 (0.46 – 6.01) | 0.43 |
| *Sarcophaga* species | Outdoor | 62 | 47 | 0.8 (0.1 – 1.5) | 1 |  |
|  | Indoor | 126 | 68 | 0.5 (0.3 – 0.7) | 0.73 (0.28 – 1.93) | 0.53 |
| *Musca domestica* | Outdoor | 62 | 14 | 0.2 (-0. – 0.5) | 1 |  |
|  | Indoor | 126 | 25 | 0.2 (0.1 – 0.3) | 1.00 (0.30 – 3.38) | 1.00 |
| **Rainy seasons (December – May)** | | | | | | |
| *Chrysomya putoria* | Outdoor | 31 | 72 | 2.3 (0.0 – 4.6) | 1 |  |
|  | Indoor | 129 | 390 | 3.0 (1.3 – 4.7) | 2.25 (0.74 – 6.79) | 0.15 |
| *Sarcophaga* species | Outdoor | 31 | 19 | 0.6 (-0.2 – 1.3) | 1 |  |
|  | Indoor | 129 | 134 | 1.0 (0.6 – 1.4) | 3.77 (0.78 – 18.13) | 0.10 |
| *Musca domestica* | Outdoor | 31 | 19 | 0.6 (-0.2 – 1.4) | 1 |  |
|  | Indoor | 129 | 167 | 1.3 (0.3 – 2.2) | 1.80 (0.43 – 7.56) | 0.42 |
| **Dry & Wet seasons (January 2022 – December 2023)** | | | | | | |
| *Chrysomya putoria* | Outdoor | 93 | 137 | 1.5 (0.6 – 2.4) | 1 |  |
|  | Indoor | 255 | 533 | 2.1 (1.2 – 3.0) | 1.28 (0.57 – 2.87) | 0.55 |
| *Sarcophaga* species | Outdoor | 93 | 66 | 0.7 (0.2 – 1.2) | 1 |  |
|  | Indoor | 255 | 202 | 0.8 (0.6 – 1.0) | 0.61 (0.23 – 1.60) | 0.32 |
| *Musca domestica* | Outdoor | 93 | 33 | 0.4 (0.1 – 0.7) | 1 |  |
|  | Indoor | 255 | 192 | 0.8 (0.4 – 1.2) | 1.39 (0.59 – 3.29) | 0.46 |

**Supplementary Figure 1** Kitchens in study houses. Where a is a Star home, b is a Star home kitchen and c is a traditional kitchen or cooking area.


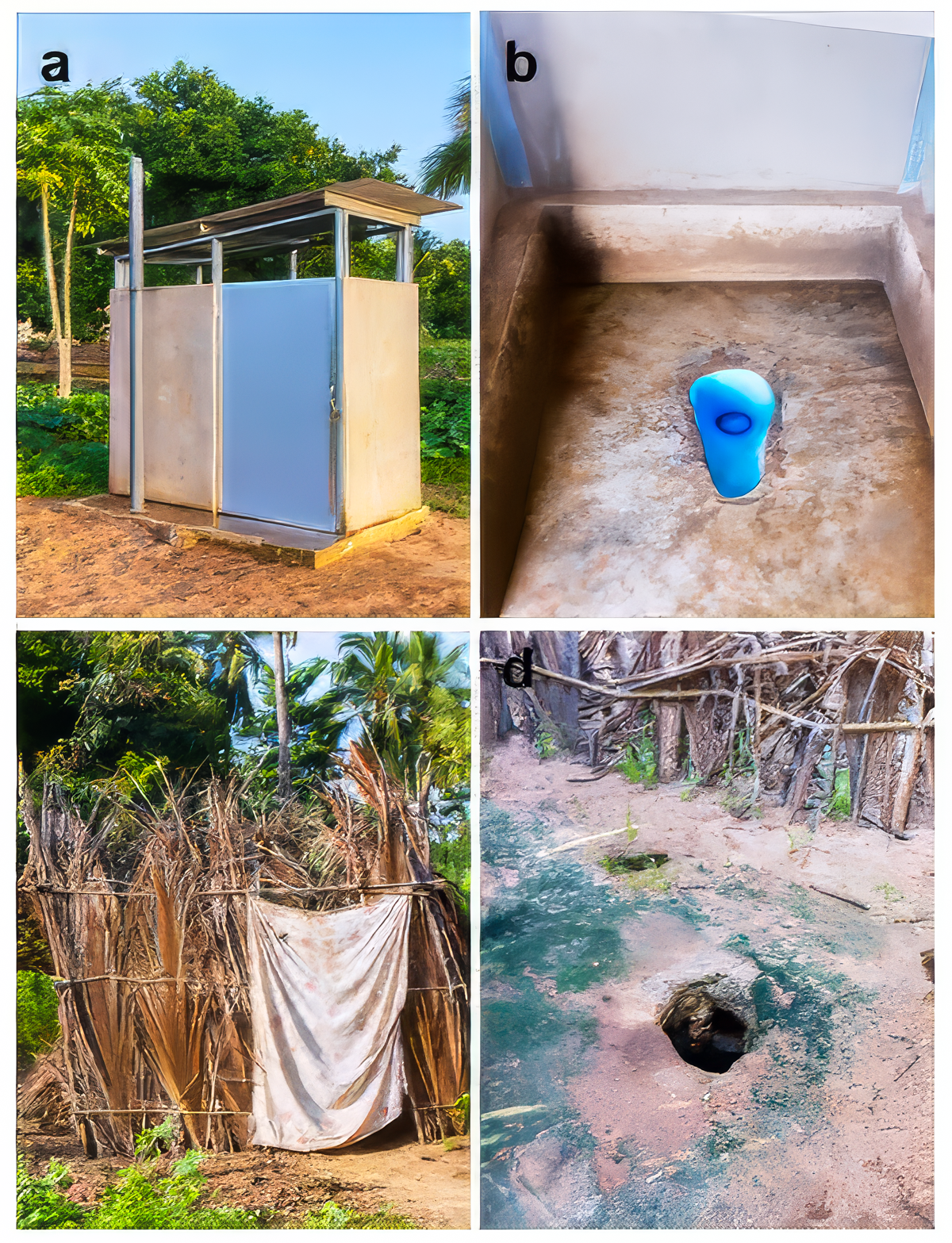


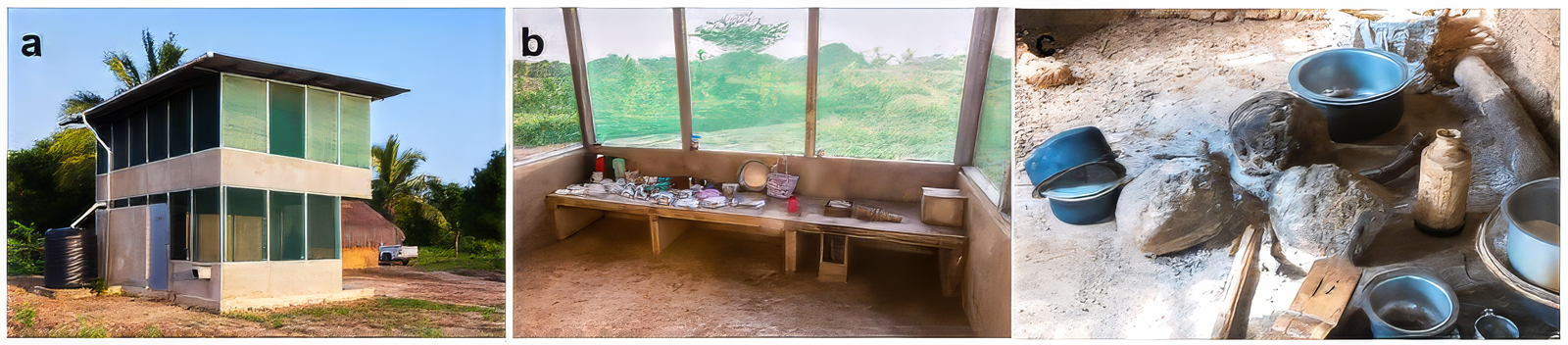


**Supplementary Figure 2** Toilets of study houses. Where a is the exterior of a Star home latrine, b the interior of a Star home latrine, c is an external view of a traditional latrine and d the interior of a traditional latrine.

**Supplementary Figure 3** Odour-baited fly trap. Where a is the external view, b shows the conical openings of a trap and c shows the rain cover for the trap.


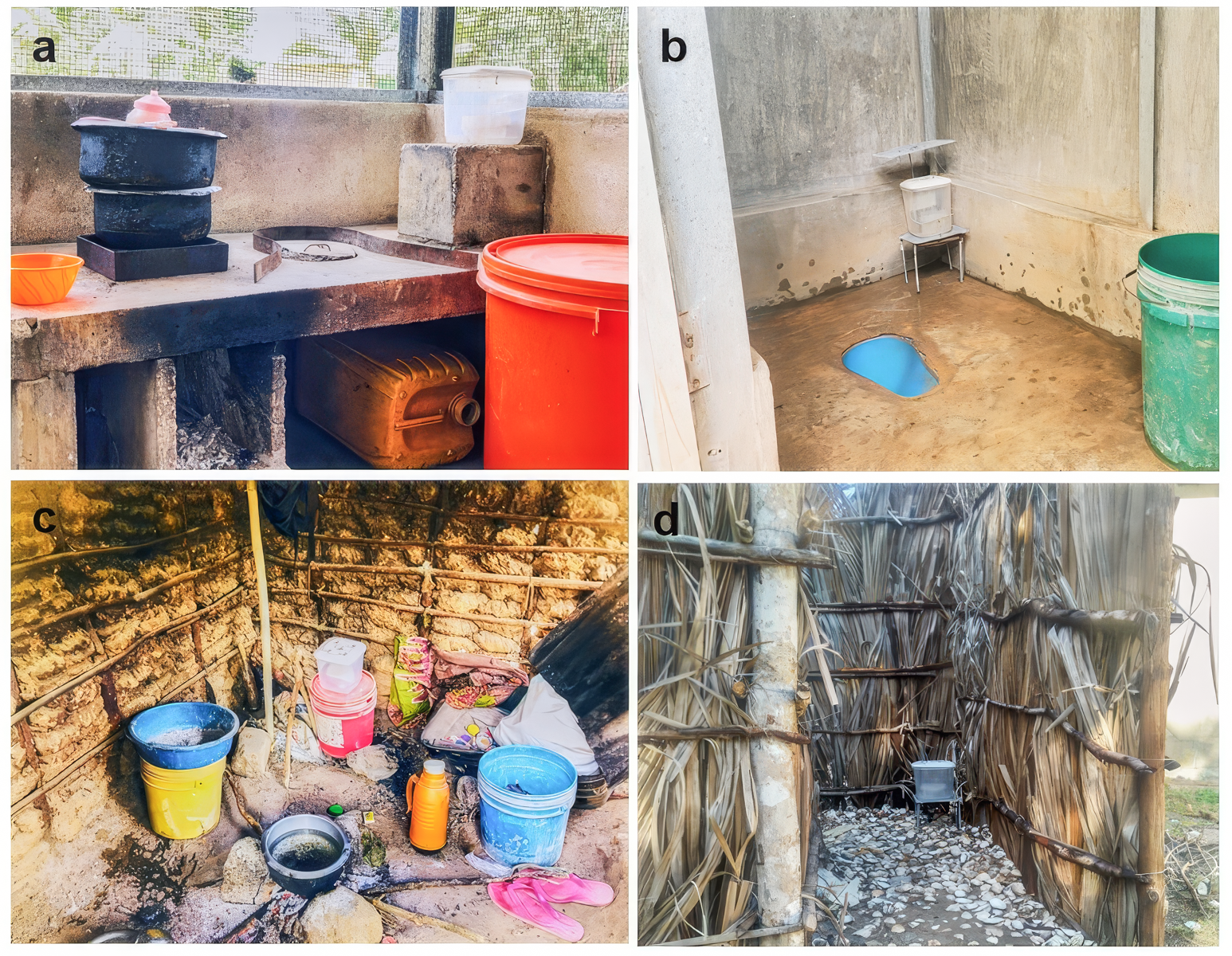


**
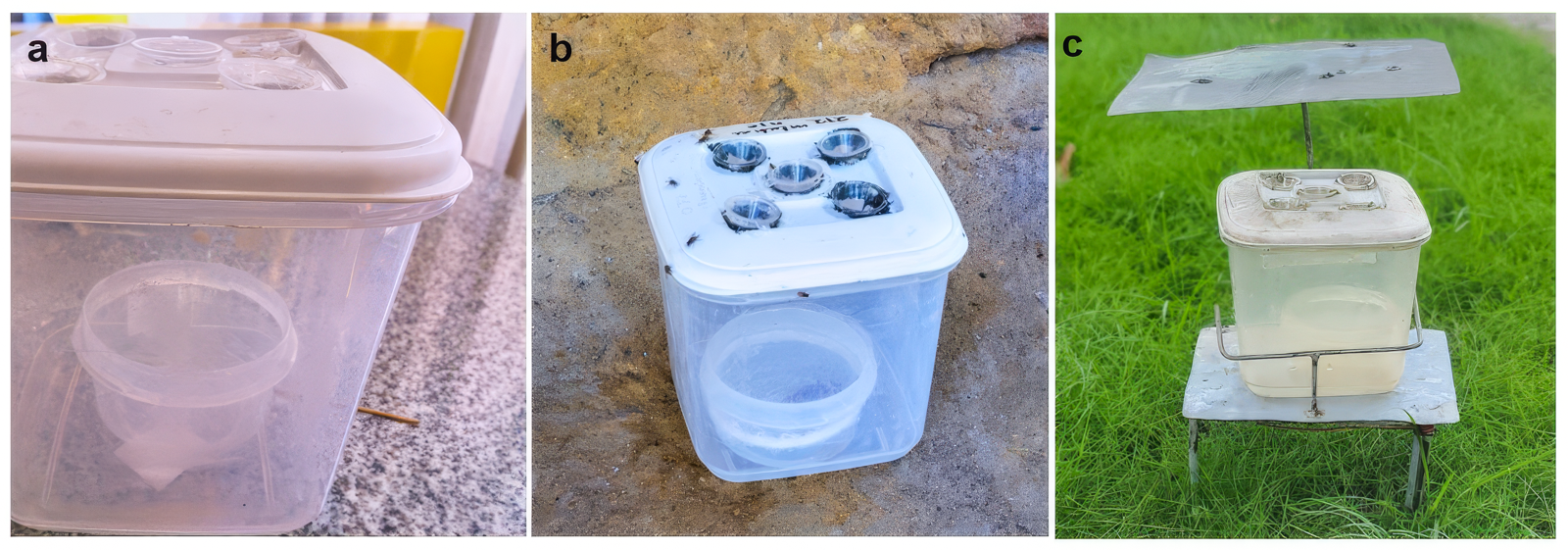
**

**Supplementary Figure 4** Position of fly traps in a) Star home kitchen and b) Star home toilet, and in a c) traditional kitchen and d) traditional toilet.


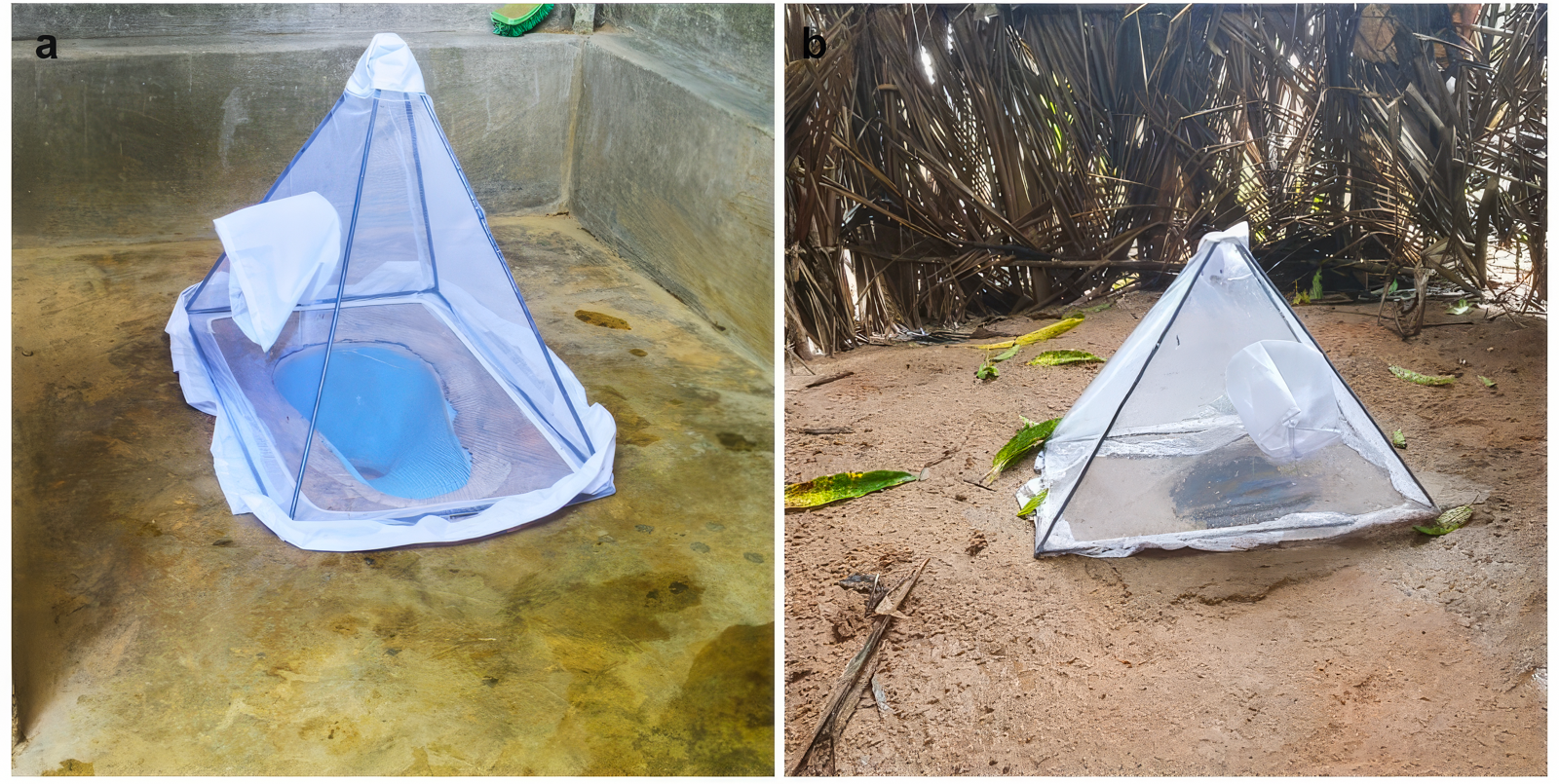


**Figure 5** Emergence traps used for collecting flies emerging from study toilets a) Star home, b) traditional house.
